# Localization of epileptic seizure focus by computerized analysis of fMRI recordings

**DOI:** 10.1186/s40708-020-00114-0

**Published:** 2020-10-31

**Authors:** Rasoul Hekmati, Robert Azencott, Wei Zhang, Zili D. Chu, Michael J. Paldino

**Affiliations:** 1 Department of Mathematics, University of Houston, Houston, TX USA; 2 Department of Radiology, Texas Children’s Hospital, Houston, TX USA; 3 Outcomes & Impact Service, Texas Children’s Hospital, Houston, TX USA; 4 Department of Radiology, Baylor College of Medicine, Houston, TX USA

**Keywords:** Time series, Deep learning, Mutual information, fMRI, Epilepsy, Seizure focus

## Abstract

By computerized analysis of cortical activity recorded via fMRI for pediatric epilepsy patients, we implement algorithmic localization of epileptic seizure focus within one of eight cortical lobes. Our innovative machine learning techniques involve intensive analysis of large matrices of mutual information coefficients between pairs of anatomically identified cortical regions. Drastic selection of pairs of regions with biologically significant inter-connectivity provides efficient inputs for our multi-layer perceptron (MLP) classifier. By imposing rigorous parameter parsimony to avoid overfitting, we construct a small-size MLP with very good percentages of successful classification.

## Introduction

Epilepsy is a common brain disorder that affects 6 out of 1000 children in the United States [36, 52]. Although medication is the mainstay of treatment for these patients, seizures are resistant to anti-epileptogenic drugs in approximately 30% of children. Such medically refractory epilepsies are frequently “focal”, defined as originating habitually from a relatively localized region of the brain. In appropriately selected patients, resection of this localized seizure origin can be curative. Despite the promise of this approach, surgical outcomes remain highly variable, even in optimal candidates [6, 7, 17, 24]. Emerging studies motivated by these inconsistencies have demonstrated that pediatric focal epilepsy is not a localized disorder, but rather a disease of large-scale, distributed neural networks [40]. Furthermore, rather than resulting from abnormal activity generated by one or more abnormal foci, the primary organization of seizure origin occurs within a functionally and anatomically connected set of brain regions—a seizure network [2]. Although the exact relationship between ictal dynamics and the inter-ictal seizure network remains to be defined, pathologic connectivity within the seizure network likely contributes to the transition between inter-ictal and ictal states [40, 43]. In other words, the capacity of this network for seizure generation results not only from abnormal hyperactivity within intrinsically dysplastic neural elements, but also from abnormal interactions between otherwise “normal” brain predisposed by pathologic *interictal* connectivity within the seizure network [38, 43]. In some cases this network may be relatively restricted in extent; at other times, it encompasses neuronal populations dispersed throughout multiple distant brain structures [40, 50]. Regardless of extent, failure to sufficiently resect or disconnect the seizure network may be associated with continued seizures after removal of the seizure focus, thereby contributing to surgical failure [1, 18]. At the current time, there is no modality that can accurately map the extent of the seizure network.

Advances in MRI combined with mathematical network approaches have demonstrated great potential to study brain networks non-invasively in a variety of populations, including children with focal epilepsy [4, 5, 20, 21, 41, 45–47]. Resting-state functional MRI is one method by which connectivity in the brain can be measured. This sequence quantifies the blood oxygen level dependent (BOLD) signal over time as an indirect measure of neuronal activity [3, 25]. Functionally connected elements of the brain exhibit similar spontaneous BOLD signal fluctuations at rest [3], which enables one to capture interactions between brain regions in vivo. Within the network framework, the brain is represented as a graph, a mathematical construct consisting of a set of nodes connected by edges [12, 14, 26, 28, 30, 41, 42]. The nodes are defined as anatomical regions of the brain; the edge between each pair of nodes is estimated as the magnitude of association between their BOLD time series [14, 23, 29, 34, 53]. Studies based on such resting state functional network constructs have consistently shown global aberrations of the epileptic brain from normal; they have also demonstrated that network features have the potential to predict clinically relevant aspects of brain function in children with epilepsy [22, 26, 28, 30, 32, 38, 44, 55]. However, little data exist regarding the use of neuroimaging-defined network models to identify the seizure network in an individual child.

The goal of this study was to assess the potential of MR imaging-defined networks to depict seizure networks. Specifically, we trained an artificial neural network (ANN) to identify the location of seizure origin given access only to features derived from a resting-state functional network. We measured the accuracy of the learning algorithm against the lobe of seizure origin determined at multidisciplinary epilepsy surgery conference. We selected this reference standard—which relies on the consensus of multiple objective tests as well as the clinical expertise from multiple disciplines—as an accurate and reliable assessment of the lobe of seizure origin [18, 56]. Identification of the lobe of seizure origin can be considered an initial step toward the ultimate validation of the potential for resting-state functional networks to visualize seizure networks in children with focal epilepsy.

## Methods

### Patients

The HIPAA-compliant study was approved by the local institutional review board. Informed consent was waived for this retrospective study. Patient medical records were retrospectively reviewed to identify patients with the following inclusion criteria: (1) pediatric age group (21 years of age or younger); (2) a clinical diagnosis of focal epilepsy; (3) an available 3-T MR imaging of brain, including a resting-state fMRI sequence; and (4) lobe of seizure origin identified at multidisciplinary epilepsy conference. This determination relies on the consensus of multiple objective tests (including MRI and EEG) as well as the clinical expertise from multiple disciplines (including Neurology, Neurosurgery, and Neuroradiology) [19, 20]. Images were performed from January 2012 to December 2017. Forty-six patients were included. Lobe diagnoses for the cohort are presented in Table 1.

**Table 1:** Lobe diagnosis of the cohort

Lobe	Frequency
Group 1—right frontal (RF)	10
Group 2—left frontal (LF)	8
Group 3—right temporal (RT)	7
Group 4—left temporal (LT)	11
Group 5—others: right and left parietal + right and left occipital (C5)	6

The 5 classes are not perfectly disjoint because 4 patients were ambiguously diagnosed by clinicians and thus belong to 2 classes simultaneously (RT/C5, RF/RT, LF/LT, LT/RF)

### MR imaging

All imaging was performed on a 3-T Achieva system (Philips, Andover, Massachusetts) with a 32-channel phased array coil. The following sequences were obtained: 1. Structural images: sagittal volumetric T1-weighted images [repetition time (TR)/echo time (TE): 7.2/2.9 ms; 1 acquisition; flip angle: 7°, inversion time: 1100 ms; field of view (FOV): 22 cm; voxel size (mm): 1 × 1 × 1)]. 2. Resting state fMRI: axial single-shot echo planar imaging (EPI) fMRI [TR/TE (ms): 2000/30; flip angle: 80°; 1 acquisition; FOV: 24 cm; voxel (mm): 3 × 3 × 3.75; 300 volumes (duration: 10 min)] performed in the resting state. All images were visually inspected for artifacts, including susceptibility and subject motion.

### Image processing and analysis

The processing pipeline was implemented using MATLAB scripts (version 7.13, MathWorks, Inc) in which adapter functions were embedded to execute FreeSurfer reconstruction (version 5.3.0) and several FMRIB Software Library (FSL) suite tools [39]. Details regarding this pathway have been previously described [26, 28, 30]. A brief summary is provided here.

The reference space was created from images of one patient in our database, who had no visible abnormality and with optimal registration to MNI space [10]. Structural imaging data for each patient were aligned to a standard reference template (MNI152) using FSL’s non-linear registration algorithm [37, 39]. Nodes in the network were defined on the template according to parcellation of whole-brain gray matter as follows: First, FreeSurfer reconstruction of cerebral cortical surfaces was performed on the T1 structural image. This processing stream includes motion correction, skull stripping, intensity normalization, segmentation of white matter and gray matter structures, parcellation of the gray matter and white matter boundary, and surface deformation following intensity gradients which optimally place the gray matter/white matter and gray matter/cerebrospinal fluid borders [8, 9]. The pial and gray-white surfaces were visually inspected using the Freeview software for accurate placement.

Next, a self-developed MATLAB program was applied to the FreeSurfer output to further subdivide the 148 standard gray matter regions according to their surface area [26–30]. During this process, each region was iteratively divided into two new regions of equal size until the surface area of each parcel (as defined on the FreeSurfer gray-white surface mesh) was less than a size threshold of 350 mm^2^. The final parcellation contained 780 nodes. Each surface parcel was then converted into a volume mask of gray matter at that region to form a node on the network. All nodes defined in reference space were transformed into each individual patient’s space by applying the non-linear transformation matrix (12 degrees-of-freedom) obtained during registration.

The first 5 volumes in each resting state functional data were removed to allow magnetization to reach equilibrium. Standard preprocessing and independent component analysis (ICA) of the functional data sets was performed using FSL MELODIC [39], consisting of motion correction, interleaved slice timing correction, brain extraction, spatial smoothing with a Gaussian kernel full width at half maximum of 5 mm, and high pass temporal filtering equivalent to 100 s (0.01 Hz). Affine boundary-based registration as implemented in FSL FLIRT was then used to align the pre-processed functional image volumes for each patient to that individual’s structural T1 dataset using linear registration [19]. The inverse transformation matrix was calculated in this step and subsequently used to transform all masks from structural to functional space. Mean BOLD-signal time series were then computed for each node.

#### Mutual information between discrete random variables

Mutual information $MI(X,Y)$ between two random variables $X$ and $Y$ quantifies the amount of information brought by the knowledge of $X$ for the prediction of$Y$. This quantification is symmetric in $X$ and $Y$, and is based on the concept of conditional entropy. When $X$ and $Y$ can take only a finite number of values denoted ${x}_{k}$ and ${y}_{m}$, their marginal and joint probability distributions are:

$${p}_{k}=Pty\left(X={x}_{k}\right); {q}_{m}=Pty\left(Y={y}_{m}\right); {r}_{k,m}=Pty\left(X={x}_{k},Y={y}_{m}\right).$$

Then the entropies of $X,Y$, and $Z=(X,Y)$ are given by:

$$H\left(X\right)=-\sum_{k}{p}_{k}\mathrm{log}\left({p}_{k}\right)\ge 0; H\left(Y\right)=-\sum_{m}{q}_{m}\mathrm{log}\left({q}_{m}\right);$$

$$H\left(X,Y\right)=-\sum_{k,m}{r}_{k,m}\mathrm{log}\left({r}_{k,m}\right).$$

The mutual information between $X$ and $Y$ is then given by:

$$MI\left(X,Y\right)=MI\left(Y,X\right)=H\left(X\right)+H\left(Y\right)-H(X,Y)\ge 0,$$

where $MI\left(X,Y\right)=0$ if and only if $X$ and $Y$ are independent. When $X$ and $Y$ have a bivariate Gaussian distribution, the mutual information $MI\left(X,Y\right)$ is also computable as an explicit increasing function of the squared correlation:

$$MI\left(X,Y\right)=-\frac{1}{2}\mathrm{log}\left(1-{cor\left(X,Y\right)}^{2}\right).$$

However, in the non-Gaussian case, this formula is not valid. Mutual information can offer major advantages over correlation, especially in the pathologic brain [33, 35, 54]. Mutual information has remarkable invariance property which we now recall. For any random variables $(U,V)$ and any *strictly increasing functions* $f$ and $g$, the random variables $X=f(U)$ and $Y=g(V)$ verify:

$$MI\left(X,Y\right)=MI\left(f(U),g(V)\right)=MI\left(U,V\right).$$

In this formula, $f$ and $g$ can be *non-linear* functions. This MI invariance property is not satisfied by correlations, since $Cor(U,V)$ and $Cor(f(U),g(V))$ are generally different unless $f$ and $g$ are both linear functions. In the study of fMRI recordings of brain activity, the strong functional invariance of mutual information offers an interesting advantage, which we now outline.

#### Advantages of mutual information to estimate cortex connectivity

Denote our $N=780$ cortex parcels by ${CP}_{1},{CP}_{2}, \dots , {CP}_{N}$. For any two parcels ${CP}_{i},$ and ${CP}_{j},$ brain activity recordings by fMRI provide, after standard pre-treatments, two sequences of *n* = 295 recordings:

$$S\left(i\right)=[{Y}_{i}\left(1\right), {Y}_{i}\left(2\right),\dots ,{Y}_{i}\left(n\right)]$$ and $$S\left(j\right)=\left[{Y}_{j}\left(1\right), {Y}_{j}\left(2\right),\dots ,{Y}_{j}\left(n\right)\right],$$

which can be considered as random samples of $n$ observations for two random variables ${Y}_{i}$ and ${Y}_{j}$. To evaluate the level of neural interaction between the two parcels $i$ and $j$, many publications use the correlations ${Cor(Y}_{i},{Y}_{j})$ or their absolute values, which can easily be estimated from the data sequences $S(i)$ and $S(j)$*.* In this paper, we have preferred instead to use the same data $S(i)$ and $S(j)$ to estimate the mutual information ${MI(Y}_{i}{,Y}_{j})$. We now indicate why this choice has a strong pragmatic and theoretical advantage over the use of ${Cor(Y}_{i},{Y}_{j})$. Indeed, the actual values ${Y}_{i}(n)$ and ${Y}_{j}(n)$ generated by fMRI recordings after pretreatment are essentially of the form:

$${{Y}_{i}\left(n\right)=f[BOL}_{i}(n)]$$ and

$${{Y}_{j}\left(n\right)=g[BOL}_{j}(n)],$$

where at time *n*, the numbers ${BOL}_{i}(n)$ and ${BOL}_{j}(n)$ represent the BOLD (blood oxygen level dependent) signals for the two parcels ${CP}_{i}$ and ${CP}_{j}$*.* Here, the functions $f(u)$ and $g(v)$ are two *increasing* functions of $u$ and $v$, which are essentially *unknown* to the experimenter because they are determined by many variable factors such as the actual hardware settings of the fMRI acquisition modalities, the meta-parameters of the fMRI signal reconstruction software, the geometric positions of ${CP}_{i}$ and ${CP}_{j}$ within the cortex, etc. Hence, contrary to the actually observed data sequences $S(i)$ and $S(j)$, the two BOLD signal sequences $BS\left(i\right)=[{BOL}_{i}\left(1\right), \dots , {BOL}_{i}\left(n\right)]$ and $BS\left(j\right)=[{BOL}_{j}\left(1\right), \dots , {BOL}_{j}\left(n\right)]$ are in fact not directly observable. Consider the sequences $BS\left(i\right)$ and $BS\left(j\right)$ as random samples from two random variables ${BOL}_{i}$ and ${BOL}_{j}$. Since the sequences $S\left(j\right)$ and $BS\left(j\right)$ are linked by the function $f$, the observable random variable ${Y}_{i}$ is of the form ${Y}_{i}=f({BOL}_{i})$. Similarly, ${Y}_{j}$ is of the form ${Y}_{i}=g({BOL}_{j})$. Most changes in the instrumental settings of the fMRI hardware may modify $f$ and $g$ in an unknown way. Moreover, if one changes the parcels, the functions $f$ and $g$ will also be modified. Fortunately, due to the functional invariance of mutual information mentioned above, we have:

$${MI(BOL}_{i},{BOL}_{j})=MI(f\left({BOL}_{i}\right),g\left({BOL}_{j}\right)=MI({Y}_{i},{Y}_{j})$$

for arbitrary increasing functions $f$ and $g$. By contrast, ${Cor(Y}_{i},{Y}_{j})$ and ${Cor(BOL}_{i},{BOL}_{j})$ can generally be quite different unless $f$ and $g$ are linear functions, which is quite unlikely in this context. So to estimate cortex connectivity by quantifying the strength of pairwise interactions between the BOLD signals ${BOL}_{i}$, ${BOL}_{j}$ at parcels ${CP}_{i}$ and ${CP}_{j}$, the mutual information $MI({Y}_{i},{Y}_{j})$ will be more stable and more relevant than the correlation $Cor({Y}_{i},{Y}_{j})$. Consistent with theoretical superiority, the real-world advantages of MI have been documented in this setting [35, 54].

#### Numerical computation of mutual information matrix

As above, we expect strong values of ${MI}_{i,j}=MI\left({Y}_{i},{Y}_{j}\right)$ to detect the frequent presence of simultaneous high blood oxygen levels ${BOL}_{i}$ and ${BOL}_{j}$ within cortex parcels ${CP}_{i}$ and ${CP}_{j}$, a phenomenon indicative of strong neuronal interactivity between ${CP}_{i}$ and ${CP}_{j}$. Each observed time series ${Y}_{i}\left(t\right)$ involves *n* = 295 observations of the continuous random variable ${Y}_{i}$, we discretize each ${Y}_{i}$ to transform it into a random variable taking only 5 values. To this end we first compute 6 quantiles of the $n$ observations ${Y}_{i}\left(t\right)$, at levels 0, 20, 40, 60, 80, 100%. This subdivides the range of ${Y}_{i}$ into 5 intervals ${[Z}_{1},\dots ,{Z}_{5}]$ with midpoints ${[z}_{1},\dots ,{z}_{5}],$ and whenever ${Y}_{i}\left(t\right)$ falls in ${Z}_{k}$, we replace ${Y}_{i}\left(t\right)$ by $\widehat{{Y}_{i}}(t)={z}_{k}$. Then we approximate ${MI}_{i,j}$ by $MI(\widehat{{Y}_{i}},\widehat{{Y}_{j}})$.

**Fig. 1:**
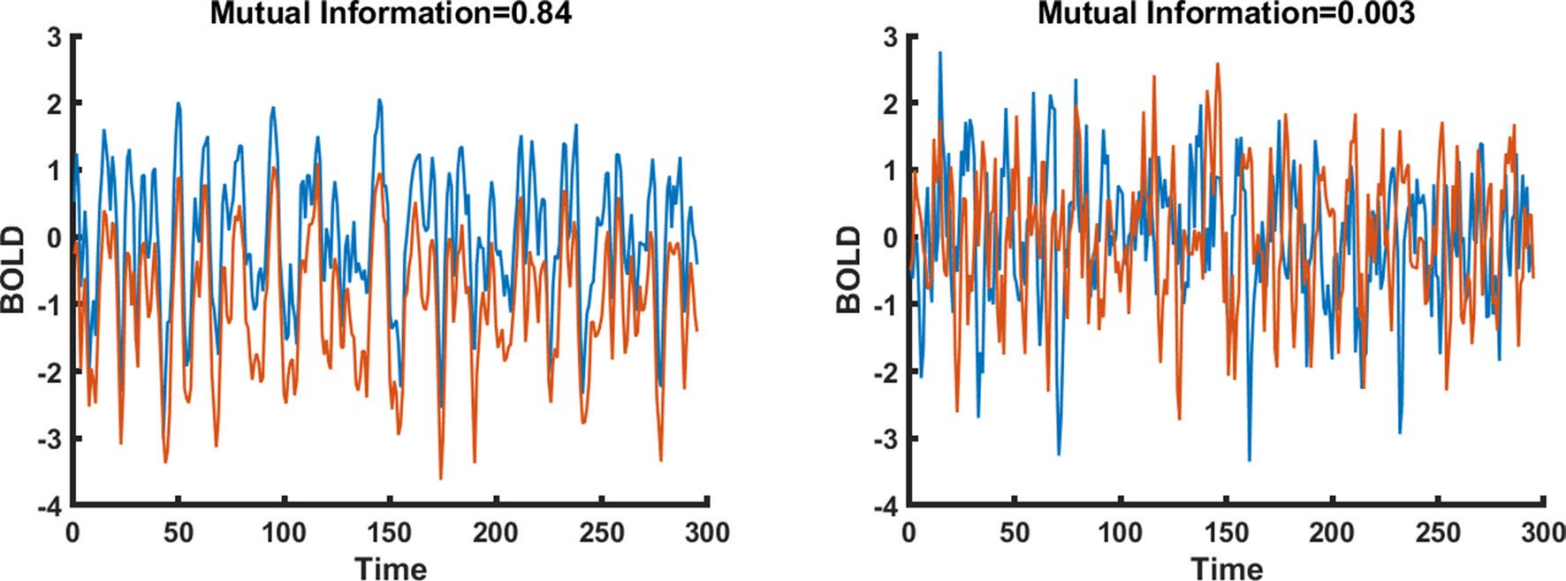
BOLD time courses for two parcels with high MI (left-hand fig.) and low MI (right-hand fig.)

To evaluate the accuracy of our mutual information estimates, we have applied a variant of the “Jackknife resampling” technique by randomly taking out 10% of time points (29 time points) and re-calculating MI on the new data set. This process has been repeated 1000 times and the absolute error of MI estimation has been calculated. For 60% of the mutual information matrix coefficients ${MI}_{i,j}$, the relative estimation error on ${MI}_{i,j}$ is inferior to 10%. The use of 20 bins instead of 5 bins to generate the discretized ${Y}_{i}\left(t\right)$ tends to double the average errors of estimation for the ${MI}_{i,j}$, as can be expected from theory. Indeed, our exploration of more detailed discretization, and even optimized discretization, has shown that using 5 bins of equal frequency 20% for each ${Y}_{i}$ was quite effective in our context. Figure 1 displays two nodes whose BOLD time courses have high MI and another two nodes whose time courses have low MI.

#### Mutual information between pairs of cortex subregions

For each patient, there are roughly 780 parcels of size 350 mm^2^ and for each parcel one time series with 295 recordings. We characterize the cortex connectivity of each patient by the symmetric 780 × 780 mutual information matrix ${MI}_{i,j}$. The coefficients of this MI matrix provide a very large number of features $\cong {780}^{2}/2$ for each patient [31]. To reduce the number of features we have developed and implemented an innovative multi-scale analysis of these MI matrices.

For any cortex subregion $A$, denote $L(A)$ the list of ${n}_{A}$ cortex parcels contained in $A$. For any two cortex subregions $A$ and $B$ generate the set $S(A,B)$ of all ${n}_{A}$ by ${n}_{B}$ mutual information coefficients ${MI}_{i,j}$ with $i$ in $L(A)$ and $j$ in $L\left(B\right).$ Let $L(A,B)$ to be the list of all elements of $S(A,B)$. We then define the mutual information $MI\left(A,B\right)=MI(B,A)$ as the 75% quantile of the list $L(A,B)$. When $MI(A,B)$ is high, at least 25% of cortex parcels pairs ${CP}_{i}$ in $L(A)$ and ${CP}_{j}$ in $L(B)$ have high ${MI}_{i,j}$ and hence are expected to have strong neural interaction.

In our definition of $MI(A,B)$, instead of the 75% quantile, we could a priori have used other quantiles of $S\left(A,B\right)$. To explore these possibilities, for each patient and each mutual information coefficient ${MI}_{i,j}$, we have computed the Relative Standard Error of estimation ${RES(A,B)}$ for ${MI(A,B)}$. These results show that ${RES(A,B)}$ becomes fairly small when ${MI}_{i,j}$ lies between the 70%-quantile and 90%-quantile of the patient’s MI matrix coefficients, so that these ${MI(A,B)}$ can be considered as quite robustly estimated. Moreover, for regions $A$ and $B$ of small or only moderate sizes, the high quantiles of all the ${MI}_{i,j}$ in the list $L\left(A,B\right)$ are of course very sensitive to possible outliers. This has led us to explore defining $MI(A,B)$ by quantiles of $L\left(A,B\right)$ between 70 and 90% to define $MI\left(A,B\right)$. We have thus separately tested 4 quantiles namely 70, 75, 80, 85%. These tests showed that, using any one of these 4 possible quantiles to implement our features selection algorithm, we ended up with fairly similar performances for our neural network classifier, with a small qualitative advantage for the 75% quantile. This explains our choice for the definition of the regional MI coefficients $MI\left(A,B\right)$.

**Fig. 2:**
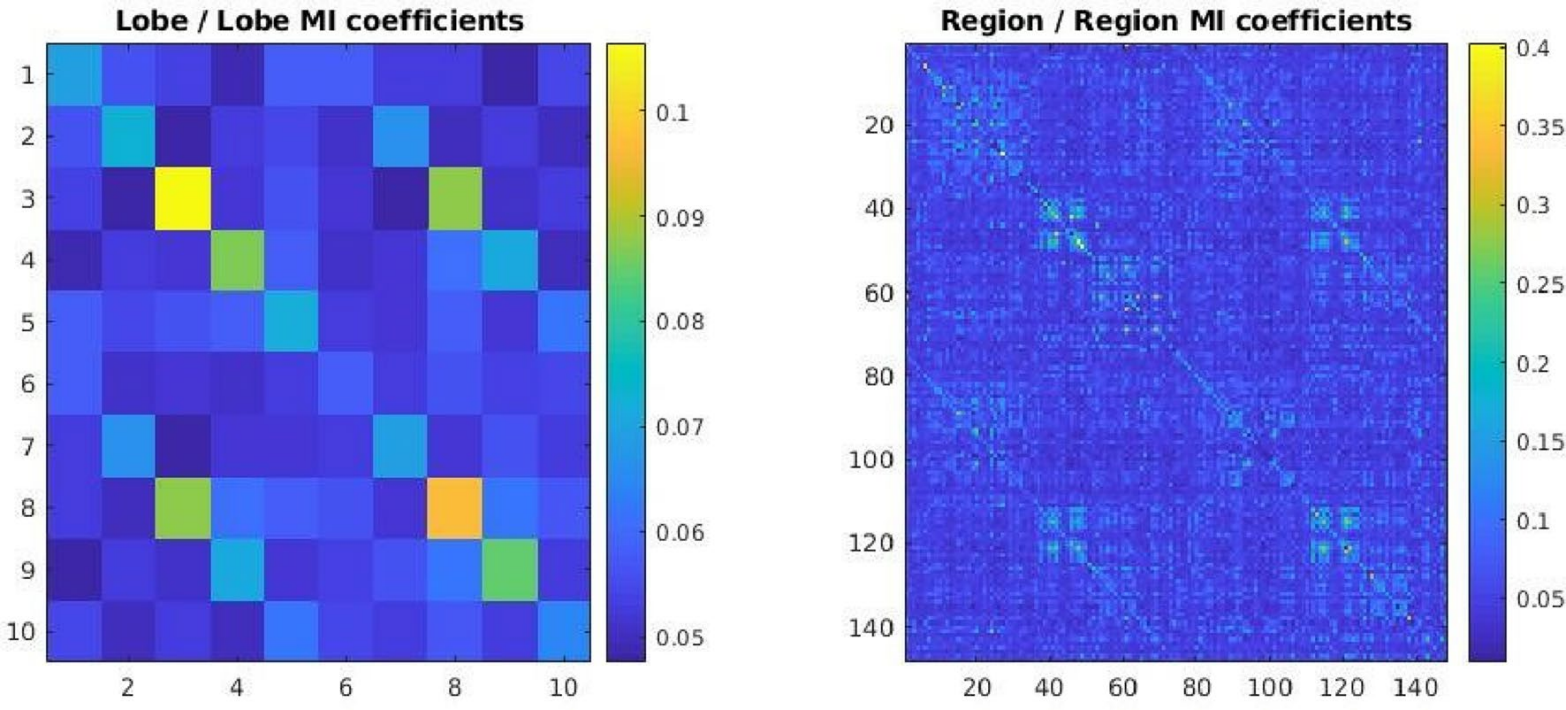
Mutual information matrices for 148 cortex regions (right-hand fig.) and for 10 cortex lobes (left-hand fig.) in a representative epilepsy patient

We then compute the coefficients $MIreg\left(m,n\right)=MI({REG}_{m},{REG}_{n})$ for any two regions in our list of 148 anatomically defined cortex regions to obtain for each patient a symmetric matrix $MIreg$ of”inter-region connectivity”. $MIreg$ has size 148 $\times$ 148, and each one of its (148*149)/2 = 11,026 distinct coefficients will provide 11,026 *potential input features* $MIreg\left(m,n\right)$ for our patient classification task. We have also studied the symmetric 10 × 10 symmetric matrices $MIlob$ of mutual information between our 10 anatomically defined cortex lobes. Figure 2 displays (for only one patient) the matrices $MIreg$ and$MIlob$.

### Automatic classification by multilayer perceptron

**Fig. 3:**
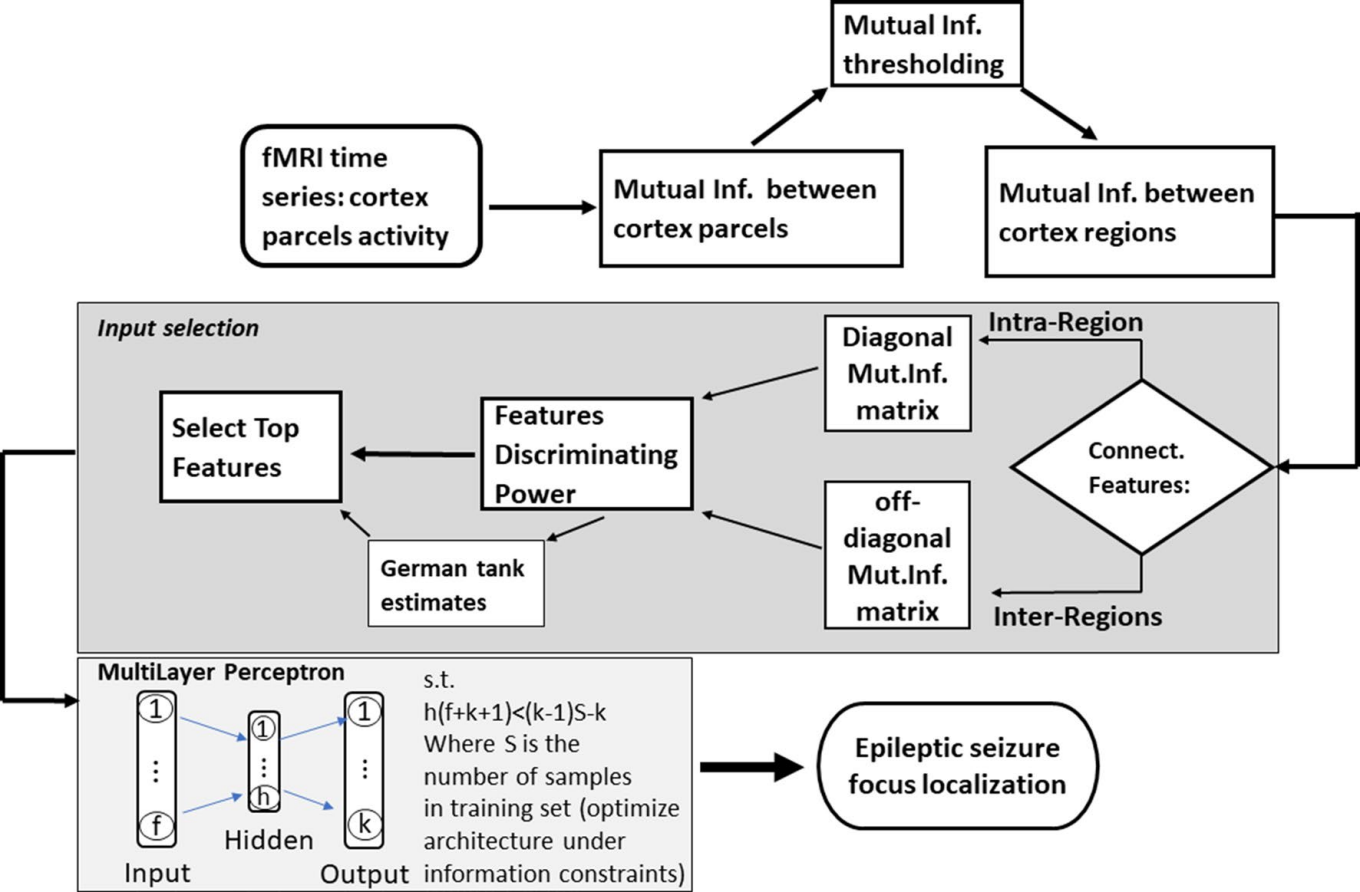
Flowchart for the pipeline

The chart in Fig. 3 shows the workflow of the pipeline from fMRI time series to epileptic seizure focus localization*.*

Here we explain the MLP architecture and its input selection procedure.

#### Architecture of our multi-layer perceptron (MLP)

To implement automatic classification of seizure origin by computer analysis of fMRI recordings of resting state brain activity, we have decided to use and evaluate Artificial Neural Networks (ANNs). Our ANNs are feed forward MLPs (multi-layer perceptrons), technically similar to the classical type of ANNs used to classify handwritten digits (MNIST data).

We first implement, as detailed further on, our feature selection algorithm which extracts a set of $f$ highly discriminating input features, selected among our 11,026 region/region mutual information coefficients $MIreg\left(m,n\right).$ For each patient, the $f$ selected input features are encoded by $f$ “neurons” which constitute the 1st layer of our MLP. The 2nd layer (called the hidden layer) contains *h* neurons, with all-to-all “synaptic” links between input layer and hidden layer. Each hidden neuron has a Sigmoid Response Function combining the $f$ input features and using $f+1$ unknown parameters ($f$ weights and *one* threshold). The 3rd layer of our MLP contains $k=5$ neurons (1 neuron for each one of our $k$ classes of patients). Each one of these $k$ neurons is linked to all the $h$ hidden neurons and has a Sigmoid Response function, involving $h+1$ unknown parameters. The 4th MLP layer is the output layer and contains $k$ neurons. Each output neuron is linked to all the $k$ neurons of the 3rd layer. The response function of output neuron $j$ is classically computed by the SoftMax formula, which involves no unknown parameter, and which generates the probability $p(j)$ that the current $f$ inputs come from a patient belonging to class $CL(j)$.

#### Parameters parsimony constraints for the MLP inputs

Most MLPs trained on very large training data sets, such as the MNIST data, have many layers successively generated by deep learning, and a quite large number of neurons. But here, given the moderate number of diagnosed patients in our data set, we have instead focused on implementing a radical reduction of the number of weights and thresholds to be learned during MLP training, in order to enforce a robust learning of synaptic weights and thresholds. Enforcing a strong parsimony for the number of unknown parameters (weights and thresholds) to be “learned” by an MLP is indeed known to *enhance the robustness* of automatic classifiers [15, 16]. Indeed, deep theoretical results of Vapnik [48] have demonstrated that this is a good recipe to perform stable automatic learning on a moderately large training set.

In such situations, feedforward neural networks (such as MLPs) are known to exhibit better generalization capacity and less overfitting when their architecture involves fewer weights. But one still wants the MLPs to achieve good classification performance, which often requires to increase the number of weights. Our approach to implement an ideal balance between these two criteria has involved a meticulous algorithmic selection of a small number of highly discriminating input features for our MLPs. Let “$f$” be the number of selected input features and “$k$” be the number of patient classes. Our MLP classifiers involve 4 successive layers of artificial neurons: an input layer of size $f$, a hidden layer of size $h$, a 3rd layer of size $k=5$, and finally an output layer of size $k$ which computes $k$ probabilities $p\left(1\right)\dots p(k)$ verifying $p\left(1\right)+\dots +p\left(k\right)=1$. The number $w$ of weights and thresholds (also called “unknown parameters”) of such an MLP is given by:

$$ w = h(f + k + 1) + k. $$

Call $W$ the vector of all $w$ unknown parameters. Each patient provides a vector $U$ of $f$ input values for our MLP, and the outputs $p\left(1\right)\dots p(k)$ computed by the MLP are all of the form $p\left(j\right)={G}_{j}(U,W)$ where ${G}_{j}$ is an explicit non-linear function of $U$ and $W$. But these functions are linearly dependent because by construction one always has ${G}_{1}+\dots +{G}_{k}=1$*.* For the *n*th training case, the input ${U}_{n}$ is known, as well as the true values ${v}_{1}(n)\dots {v}_{k}(n)$ for the $k$ MLP outputs ${p\left(1\right)\dots p(k)}$. In the coding of true outputs, for a case in class $CL(j)$, the only non-zero true output is ${v}_{j}=1$, so that ${v}_{1}+\dots +{v}_{k}=1$*.* Hence the *n*th training case yields $k$ non-linear equations satisfied by the vector $W$ of unknown parameters, namely:

$${G}_{1}\left({U}_{n},W\right)={v}_{1}\left(n\right);\dots ;{G}_{k}\left({U}_{n},W\right)={v}_{k}\left(n\right).$$

But these $k$ equations are *linearly dependent* because as seen above their sum is always equal to 1. Thus, each training case yields in fact only $(k-1)$ non-linear independent constraints to be satisfied by the $w$ unknown parameters. Hence a training set of size $S$ provides $(k-1)\times S$ non-linear equations to be satisfied by $w$ unknown parameters. To avoid overfitting, one should “ideally” have $w<\left(k-1\right) S$, which yields the following dimensional constraint on “$h$” and “$f$”:

Parameters parsimony constraint $h \left(f+k+1\right)<\left(k-1\right)S-k.$

Here, we have $k=5$ patient classes and $S = 45$ because we use *leave-one-out* training. Hence, the parameters parsimony constraint on $(h,f)$ becomes here as $h \left(f+6\right)\le 175$.

#### Implementation of automatic learning

After selecting $f$ highly discriminating input features for our MLP, we also fix the size $h$ of its hidden layer, making sure that $f$ and $h$ verify the parsimony constraint $h \left(f+6\right)\le 175$. During training of this MLP, when a case is ambiguously diagnosed as belonging to 2 classes $CL(i)$ and $CL(j)$, we assign to this case MLP outputs ${v}_{i}={v}_{j}=1/2$. Automatic learning is performed by standard Gradient Descent, to minimize the average “Cross Entropy” between true outputs and MLP-computed outputs. Indeed, for classification tasks, minimizing cross entropy is generally more efficient than minimizing mean squared error.

Since our training set is of moderate size, we implement the classical leave-one-out technique. Namely, one eliminates one patient from the training set before performing automatic learning; then one evaluates by 0 or 1 the correctness of our MLP classification of this left-out patient. Final performance is the average of these evaluations over all possible choices of the left-out patient.

#### Input selection for minimal size MLP classifiers

We want to select “features” discriminating between 5 classes of patients $CL(1)$*,…*$CL(5)$ of sizes $s\left(1\right)\dots s(5)$. This requires solving at least 10 basic tasks of the type “discriminate $CL(p)$ versus $CL(q)$”. Consider any feature $F$ computable from fMRI recordings, and hence providing for each patient $PAT$ a number $F(PAT).$ For each class $CL(p),$ the $s(p)$ patients [$PAT$ 1, $PAT$ 2, …] of $CL(p)$ generate a list $V(p)$ of $s(p)$ values $F1=F\left(PAT1\right), F2=F(PAT2)$, …. The probability distribution ${\mu }_{p}(F)$ of $[F1,F2,\dots ]$ is unknown, and difficult to estimate if $s(p)$ is not very large. For feature efficiency evaluation only, we roughly approximate ${\mu }_{p}(F)$ by a uniform distribution on an unknown interval ${J}_{p}(F)$. A classical algorithm to estimate ${J}_{p}(F)$ is the “German Tank Estimate” (see Appendix), which computes first the min $m(p)$ and the max $M(p)$ of the list $V(p),$ and then extends adequately the interval $[m(p), M(p)]$.

When ${J}_{p}(F)$ and ${J}_{q}(F)$ are nearly disjoint intervals, we naturally consider that the classes $CL(p)$ and $CL(q)$ are strongly separated by feature $F$.To quantify this notion, define the *separability *$sep(J,J^{\prime})$ of two intervals *J *and *J*′ with intersection $I = J \cap J^{\prime}$ by:

$$ sep(J,J^{\prime}) = \min \left( {\frac{{\left| {J - I} \right|}}{\left| J \right|},\frac{{\left| {J^{\prime} - I} \right|}}{{\left| {J^{\prime}} \right|}}} \right), $$

where $K$ is the length of interval $K$. Then $0 \le sep(J,J^{\prime}) \le 1$, and values of $sep(J,J^{\prime})$ close to 1 mean that $J,J^{\prime}$ are nearly disjoint. Now define ${DIS}_{p,q}{(F})=sep({J}_{p}\left(F\right),{J}_{q}(F))$ as the *discriminating power* of feature $F$ to differentiate between classes $CL\left(p\right)$ and $CL\left(q\right).$

To select a small but efficient set of “$f$” input features for our MLP classifier, we start from the (148)(149)/2 mutual information coefficients $MIreg(m,n)$ at the cortex regions “scale”. For each pair $m\le n$, the “regions based” feature ${F}_{m,n}=MIreg(m,n)$ provides for each patient an indicator of the neuronal interaction level between cortex regions ${REG}_{m}$ and ${REG}_{n}$. Fix any two distinct classes $CL (p), CL (q).$ For all $(m,n)$ with $1\le m\le n\le 148$, we compute the power ${POW\left(m,n\right)=DIS}_{p,q}{(F}_{m,n})$ of feature ${F}_{m,n}$ to discriminate $CL(p)$ vs $CL(q)$. Then we rank the features ${F}_{m,n}$ by decreasing $POW(m,n)$, and select only the top two ${F}_{m,n}$ having highest discriminating power $POW(m,n)$.

## Results

### Inter-region connectivity input to MLP

**Fig. 4:**
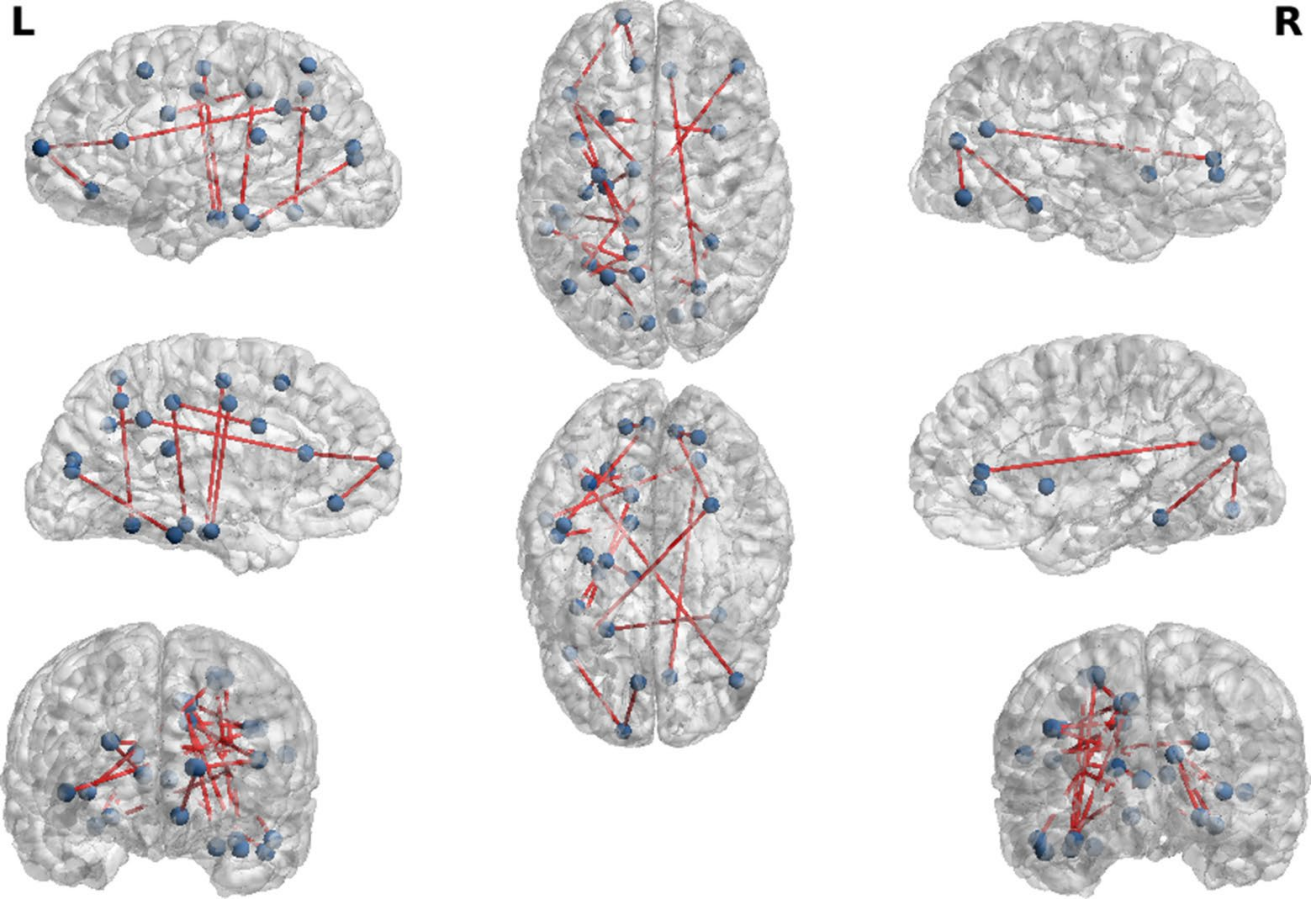
20 pairs of cortical regions with strongly discriminating inter-region connectivity coefficients

For each one of the 10 pairs $CL (p), CL (q)$ with 1 ≤ *p* < *q ≤ *5, the preceding selection algorithm selects two regions ${Reg}_{m}$, ${Reg}_{n}$. This algorithm, then, generates a set of 20 pairs of cortex regions ${REG}_{m}$*, *${REG}_{n}$ with strongly discriminating coefficients $MIreg\left(m,n\right)$ (Fig. 4). To respect the parameter parsimony constraint, we keep only a set of 18 of these Inter-region Connectivity Coefficients, to be used below as a set of 18 inputs features ${F}_{m1,n1},\dots , {F}_{m18,n18}$ for our first MLP classifier.

### Intra-region connectivity input to MLP

**Fig. 5:**
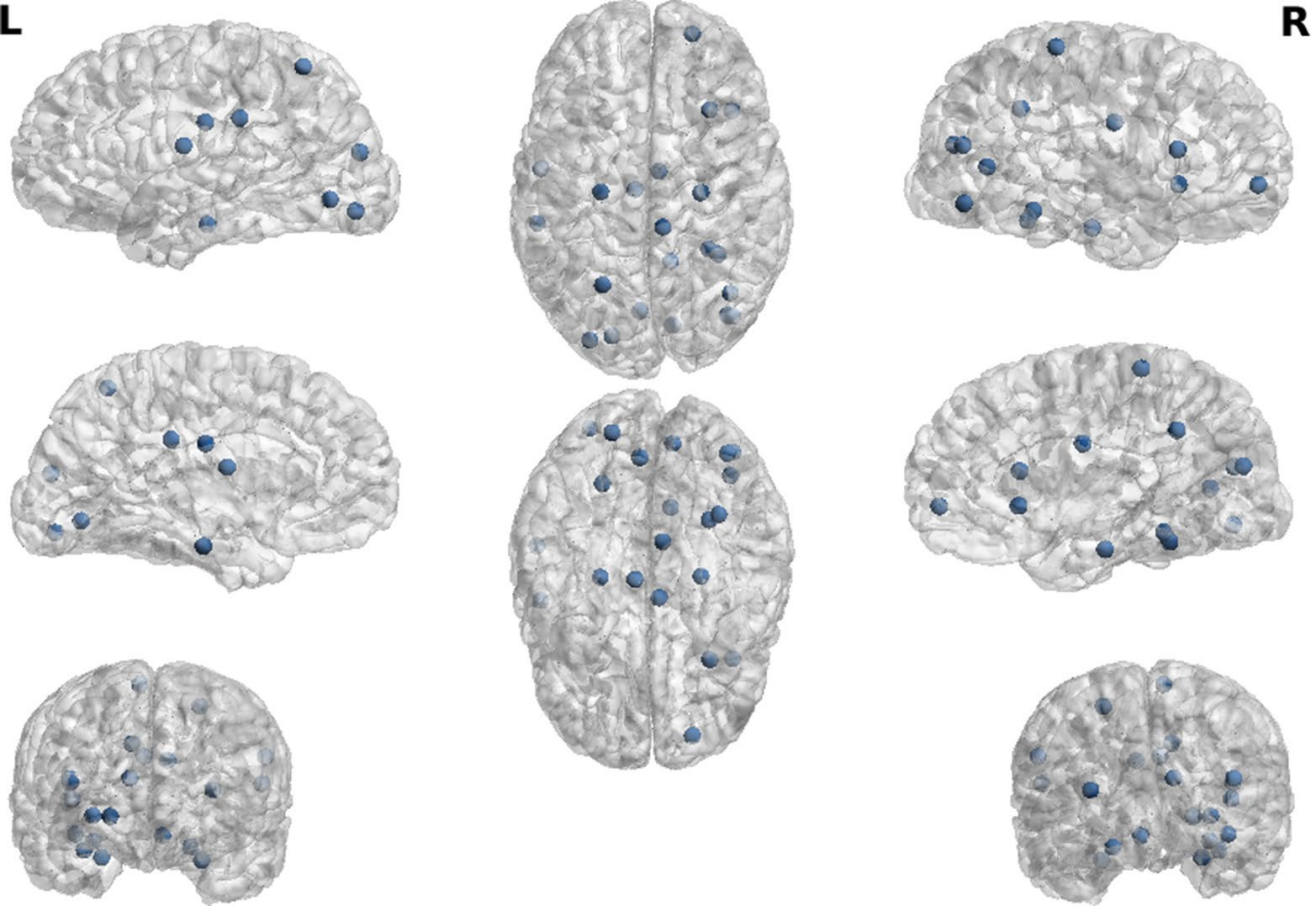
Twenty-one cortical regions with strongly discriminating intra-region connectivity coefficients

The intra-region connectivity coefficient $MIreg(m,m)$ of cortex region ${Reg}_{m}$ is an indicator of the level of neuronal interactions within ${Reg}_{m}$. The selection algorithm outlined above can detect the regions ${Reg}_{m}$ for which the feature ${F}_{m,m}=MIreg(m,m)$ has strong power $POW(m,m)$ to discriminate between at least two classes $CL(p), CL(q).$ We have thus selected a set of 21 such cortex regions, 21 intra-connectivity coefficients (Fig. 5), from which we keep only a set of 18 coefficients to be used below as input features of our second MLP classifier.

### Discriminating power for inter- and intra-region connectivity coefficients:

**Fig. 6:**
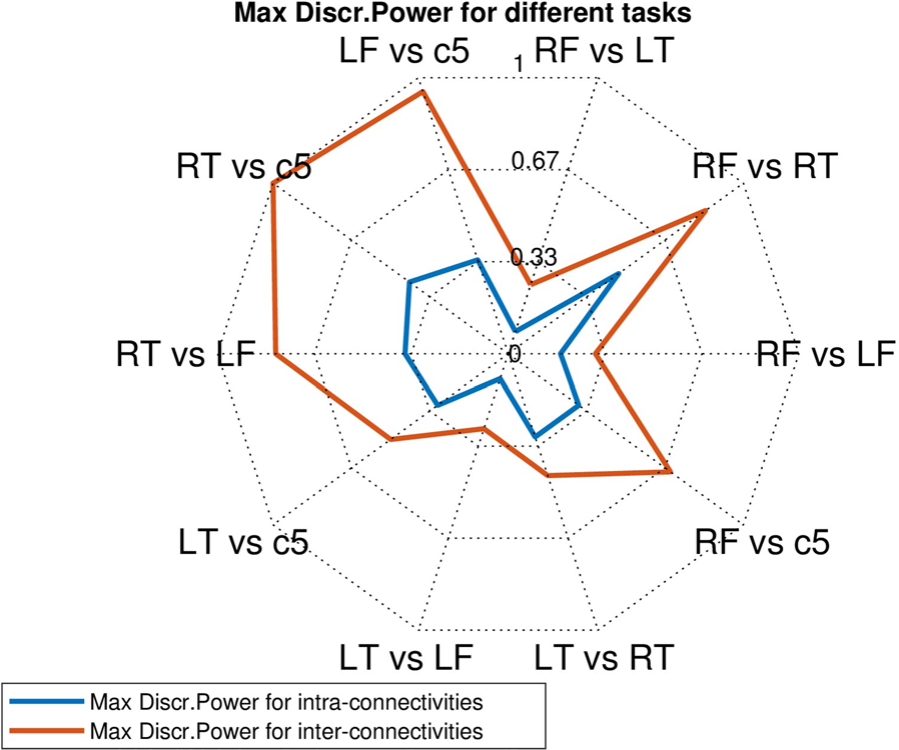
Highest power reached for the 10 basic discrimination tasks

For each one of the 10 basic discrimination tasks $CL(p)$ vs $CL(q)$ the highest discriminating power $DIS(p,q)$ reached for this task among all the connectivity coefficients $MIreg(m,n),$ is a crude indicator of “how well” the task may be solved by an efficient classifier. In Fig. 6, we display in red the ten $DIS(p,q)$ values reached by inter-connectivity coefficients and in blue the $DIS(p,q)$ values reached by intra-connectivity coefficients. Clearly the red values dominate the blue values.

### Patient classification by minimal MLP with highly discriminating inputs

#### MLP classifier based on inter-region connectivity coefficients

The set of 18 inter-connectivity coefficient $MIreg(m,n)$ selected above provides the $f=18$ inputs of our first MLP classifier, for which we impose a hidden layer size $h=7$. The 4 layers of this MLP have then sizes (18, 7, 5, 5). Our parameter parsimony equation becomes $h\left(f+6\right)=168<175$, and is hence correctly verified, again assuring a reasonable robustness of the MLP training results.

After training this small-size MLP, we evaluate its performances by leave-one-out technique. The global percentage of successful patient classifications over all 5 patient classes was 89 $\pm$ 2*.*1%. In Table 2, we display the percentage of successful classifications within each patient class. Most errors occur for discrimination between Classes RF and LF.

**Table 2:** Automatic classification accuracy in a leave-one-out design based on 18 strongly discriminating inter-connectivity coefficients at cortex regions scale

Patient class	RF	LT	RT	LF	Other
Accuracy (%)	82	90.5	100	87	90

#### MLP classifier based on 18 intra-region connectivity coefficients

The set $of$ 18 intra-connectivity coefficients $MIreg(m,m)$ selected above provides $f=18$ inputs for our second MLP classifier which has again a 4-layer architecture with dimensions (18, 7, 5, 5) (see Section 2) and hence satisfies our parsimony equation. After training this second MLP, performance evaluated by leave-one-out technique demonstrates a global percentage of successes of 88 $\pm$ 2*.*1%. Table 3 displays classification accuracy within each patient class. Results are slightly weaker than for our first MLP, particularly for Class RF and Class LF. Discrimination between these two classes generate most errors of classification.

**Table 3:** Classification accuracy in leave-one-out design based on 18 highly discriminating intra-connectivity coefficients

Patient class	RF	LT	RT	LF	Other
Accuracy (%)	70	80	100	87	100

### Patient classification by support vector machine (SVM) with highly discriminating inputs:

The set of 18 inter-connectivity/intra-connectivity coefficients $MIreg(m,n)$ selected above can be input to a multiclass SVM classifier that uses a one-vs-rest approach. Table 4 displays classification accuracy within each patient class (leave-one-out technique).

**Table 4:** Classification accuracy for SVM in leave-one-out design based on 18 highly discriminating connectivity coefficients

Patient class	RF	LT	RT	LF	Other
Accuracy (inter-connectivity)	%59	%64	%83	%78	%69
Accuracy (intra-connectivity)	%57	%62	%73	%71	%64

## Discussion

Our goal was to explore automatic classification of fMRI data in young epileptic patients to identify the origin of epileptic seizures. Given the moderate size of our data set, one challenge was to avoid using machine learning techniques involving large numbers of parameters. We have solved this challenge by introducing a rigorous “parameters parsimony principle”.

A second challenge was to identify strongly discriminating input features computable from our fMRI data. To this end, we have systematically used large matrices of mutual information coefficients between all pairs of roughly 780 time series extracted from each patient’s fMRI data. We have introduced a computable version of mutual information between any two cortex regions, as an indicator of neuronal interactions between these two regions. This led us to define and compute the discriminating power of all these ${148}^{2}/2$ inter-region connectivity coefficients.

We have then extracted one set of 18 strongly discriminating inter-region connectivity coefficients, and used them as input features for a small-size multi-layer perceptron (MLP) with 4 layers (dimensions: 18, 7, 5, 5), including a “SoftMax” terminal layer. After automatic training by a leave-one-out technique, this MLP provided 89% successful patients classification. For comparison, a standard support vector machine applied to our patient classification has yielded less than 71% successes. Our innovative development of mutual information between pairs of cortex regions, and of algorithmic selection of highly discriminating pairs of cortex regions has shown good capacity to extract useful and interpretable brain activity features from fMRI recordings. The small size of the MLP classifier we have thus constructed rigorously avoids overfitting and reaches good performance on our group of epileptic patients. To confirm these exploratory findings, we plan to test our approach on larger groups of patients.

We also designed and implemented an analysis to interpret why interactivity between two specific regions (say $A,B$) can separate two specific lobes (say ${L}_{1}$,${L}_{2}$). We found out that for almost all the patients with seizure focus in ${L}_{1}$, we can find *within* ${L}_{1}$ one or more cortex regions having strong interaction with $A$ and with $B$, while *all* subregions of ${L}_{2}$ have much weaker simultaneous interactions with $A$ and $B$. This means that $A,B$ are simultaneously excited whenever specific subregions of ${L}_{1}$ become strongly active, while hyperactivity in any subregion of ${L}_{2}$ has a weak impact on the simultaneous activity of $A$ and $B$. For example, the inter-connectivity between the regions = $A$ *“Transverse frontopolar gyri and sulci”* and = $B$ *“Vertical ramus of the* *anterior segment of the lateral sulcus”* has strong discriminating power between lobes ${L}_{1}=LF$ and ${L}_{2}=RF$ and we can actually find two subregions $U$ and $V$ of $LF$ with high interactivity with $A$:

$$MI\left(U,A\right)=0.22$$ and $$MI\left(V,B\right)=0.25$$,

while for all subregions $K$ of $RF$ one has:

$$MI\left(K,A\right)<0.12$$ and $$MI\left(K,B\right)<0.11$$.

These data suggest the potential for a similar approach to depict specific nodes that constitute important drivers of the seizure network.

Our methods are generally consistent with previous applications of network connectivity to invasive EEG. Wilke et al. observed a correlation of betweenness centrality, a network graph metric, with the location of network nodes whose resection was likely to result in seizure freedom [51]. Sinha et al. also validated a model of epileptogenicity based on connectivity against surgical outcomes in a cohort consisting largely of adults [38]. They observed a correlation between their index of epileptogenicity and the putative seizure onset zone; they also found areas of high epileptogenicity outside of the resection zone in several cases of unsuccessful surgery. Tomlinson et al. applied a network approach to a pediatric cohort with focal epilepsy [44]. They observed a significant increase in connectivity outside of the seizure onset zone; further, global synchrony (a measure of the overall strength of connectivity) within the field of invasive EEG could be used to classify patients with regard to seizure-free outcome after surgery. These studies not only reinforce the relevance of network features to seizure onset, they highlight the importance of broader areas of dysconnectivity (beyond the traditional zone of seizure onset) in achieving seizure freedom. Invasive monitoring in the form of electrocorticography (ECOG) or stereo EEG (SEEG) is gold standard for localization of the seizure onset zone. These modalities, however, are limited in their spatial sampling; large areas of the brain are left unexplored, leading to the potential for erroneous and biased conclusions [13]. Optimal use of invasive monitoring therefore requires an accurate pre-test hypothesis regarding the location and extent of the epileptogenic network. It is likely, therefore, that optimal patient outcomes would result from the addition of global network approaches to standard invasive monitoring.

## Conclusion

We developed machine learning algorithms to evaluate the connectivity obtained from resting-state fMRI in terms of differentiating the lobe of seizure origin in a pediatric cohort with focal epilepsy. These findings support the potential for neuroimaging-based network constructs to depict pathophysiologically relevant features of seizure genesis. If these approaches can be tailored to identify individual elements within the seizure network, biomarkers based on functional networks may ultimately contribute to personalized management strategies in children undergoing epilepsy surgery.

## Data Availability

The datasets generated during and/or analyzed during the current study are available from the corresponding author on reasonable request.

## Appendix: “German Tanks” estimate for uniform distributions

Let $\mu$ be a uniform probability distribution on an unknown interval = [*a*, *b*]. Let ${X}_{1},\dots ,{X}_{N}$ be $N$ independent random observations having the same probability distribution$\mu$. From these $N$ observations, one can compute the classical “German Tank” (GT) estimators $({A}_{N},{B}_{N})$ of $(a,b),$ which are unbiased and have minimal variance.

To compute the GT estimators, denote

$${U}_{N}=\mathrm{min}\left({X}_{1}, \dots , {X}_{N}\right), {V}_{N}=\mathrm{max}\left({X}_{1}, \dots , {X}_{N}\right).$$

One then has [11, 49]:

$$ A_{N} = U_{N} - (V_{N} - U_{N} )/(N - 1);B_{N} = V_{N} + (V_{N} - U_{N} )/(N - 1). $$

## Animal studies

This manuscript does not contain any studies with animals performed by any of the authors.

## References

[1] Bartolomei F, Chauvel P, Wendling F (2008). Epileptogenicity of brain structures in human temporal lobe epilepsy: a quantified study from intracerebral EEG. Brain.

[2] Bartolomei F, Lagarde S, Wendling F, McGonigal A, Jirsa V, Guye M (2017). Defining epileptogenic networks: contribution of SEEG and signal analysis. Epilepsia.

[3] Biswal BB, Van Kylen J, Hyde JS (1997). Simultaneous assessment of flow and BOLD signals in resting-state functional connectivity maps. NMR Biomed.

[4] Bullmore E, Sporns O (2009). Complex brain networks: graph theoretical analysis of structural and functional systems. Nat Rev Neurosci.

[5] Centeno M, Carmichael DW (2014). Network connectivity in epilepsy: resting state fMRI and EEG-fMRI contributions. Front Neurol.

[6] Cohen-Gadol AA, Ozduman K, Bronen RA, Kim JH, Spencer DD (2004). Long-term outcome after epilepsy surgery for focal cortical dysplasia. J Neurosurg.

[7] Cohen-Gadol AA, Wilhelmi BG, Collignon F, White JB, Britton JW, Cambier DM (2006). Long-term outcome of epilepsy surgery among 399 patients with nonlesional seizure foci including mesial temporal lobe sclerosis. J Neurosurg.

[8] Fischl B, Liu A, Dale AM (2001). Automated manifold surgery: constructing geometrically accurate and topologically correct models of the human cerebral cortex. IEEE Trans Med Imaging.

[9] Fischl B, Salat DH, van der Kouwe AJ, Makris N, Segonne F, Quinn BT (2004). Sequence-independent segmentation of magnetic resonance images. Neuroimage.

[10] Fonov V, Evans AC, Botteron K, Almli CR, McKinstry RC, Collins DL (2011). Unbiased average age-appropriate atlases for pediatric studies. Neuroimage.

[11] Goodman L (1954). Someparactical techniques in serial number analysis. J Am Stat Assoc.

[12] Grayson DS, Fair DA (2017). Development of large-scale functional networks from birth to adulthood: a guide to the neuroimaging literature. Neuroimage.

[13] Hader WJ, Tellez-Zenteno J, Metcalfe A, Hernandez-Ronquillo L, Wiebe S, Kwon CS (2013). Complications of epilepsy surgery: a systematic review of focal surgical resections and invasive EEG monitoring. Epilepsia.

[14] Hagmann P, Cammoun L, Gigandet X, Meuli R, Honey CJ, Wedeen VJ (2008). Mapping the structural core of human cerebral cortex. PLoSBiol.

[15] Hekmati R, et al (2018) Automatic classification of large sets of brain activity fMRI time series. Paper presented at the 4th International Conference on Big Data and Information Analytics, Houston.

[16] Hekmati R, et al (2018) Machine learning to evaluate fMRI recordings of brain activity in epileptic patients. Paper presented at the Q-Bio Conference, Houston.

[17] Hosoyama H, Matsuda K, Mihara T, Usui N, Baba K, Inoue Y (2017). Long-term outcomes of epilepsy surgery in 85 pediatric patients followed up for over 10 years: a retrospective survey. J NeurosurgPediatr.

[18] Jayakar P, Gaillard WD, Tripathi M, Libenson MH, Mathern GW, Cross JH (2014). Diagnostic test utilization in evaluation for resective epilepsy surgery in children. Epilepsia.

[19] Jenkinson M, Smith S (2001). A global optimisation method for robust affine registration of brain images. Med Image Anal.

[20] Kim D-J, Davis EP, Sandman CA, Sporns O, O'Donnell BF, Buss C (2016). Children's intellectual ability is associated with structural network integrity. Neuroimage.

[21] Li Y, Liu Y, Li J, Qin W, Li K, Yu C (2009). Brain anatomical network and intelligence. PLoSComputBiol.

[22] Liao W, Zhang Z, Pan Z, Mantini D, Ding J, Duan X (2010). Altered functional connectivity and small-world in mesial temporal lobe epilepsy. PLoS ONE.

[23] Magalhaes R, Marques P, Soares J, Alves V, Sousa N (2015). The impact of normalization and segmentation on resting-state brain networks. Brain Connect.

[24] Najm I, Jehi L, Palmini A, Gonzalez-Martinez J, Paglioli E, Bingaman W (2013). Temporal patterns and mechanisms of epilepsy surgery failure. Epilepsia.

[25] Ogawa S, Lee TM, Kay AR, Tank DW (1990). Brain magnetic resonance imaging with contrast dependent on blood oxygenation. Proc NatlAcadSci U S A.

[26] Paldino MJ, Chu ZD, Chapieski ML, Golriz F, Zhang W (2017). Repeatability of graph theoretical metrics derived from resting-state functional networks in paediatric epilepsy patients. Br J Radiol.

[27] Paldino MJ, Golriz F, Chapieski ML, Zhang W, Chu ZD (2016). Brain network architecture and global intelligence in children with focal epilepsy. AJNR Am J Neuroradiol.

[28] Paldino MJ, Golriz F, Chapieski ML, Zhang W, Chu ZD (2017). Brain network architecture and global intelligence in children with focal epilepsy. AJNR Am J Neuroradiol.

[29] Paldino MJ, Golriz F, Zhang W, Chu ZD (2019). Normalization enhances brain network features that predict individual intelligence in children with epilepsy. PLoS ONE.

[30] Paldino MJ, Zhang W, Chu ZD, Golriz F (2017). Metrics of brain network architecture capture the impact of disease in children with epilepsy. NeuroimageClin.

[31] Azencott R, Muravina V, Hekmati R, Zhang W, Paldino M (2019) Automatic clustering in large sets of time series. In: Contributions to partial differential equations and applications. Springer, Berlin, pp 65–75.

[32] Rajpoot K, Riaz A, Majeed W, Rajpoot N (2015). Functional connectivity alterations in epilepsy from resting-state functional MRI. PLoS ONE.

[33] Reshef DN, Reshef YA, Finucane HK, Grossman SR, McVean G, Turnbaugh PJ (2011). Detecting novel associations in large data sets. Science.

[34] Rubinov M, Sporns O (2010). Complex network measures of brain connectivity: uses and interpretations. Neuroimage.

[35] Rummel C, Abela E, Muller M, Hauf M, Scheidegger O, Wiest R (2011). Uniform approach to linear and nonlinear interrelation patterns in multivariate time series. Phys Rev E Stat Nonlin Soft Matter Phys.

[36] Russ SA, Larson K, Halfon N (2012). A national profile of childhood epilepsy and seizure disorder. Pediatrics.

[37] Simpson IJ, Cardoso MJ, Modat M, Cash DM, Woolrich MW, Andersson JL (2015). Probabilistic non-linear registration with spatially adaptive regularisation. Med Image Anal.

[38] Sinha N, Dauwels J, Kaiser M, Cash SS, Brandon Westover M, Wang Y (2017). Predicting neurosurgical outcomes in focal epilepsy patients using computational modelling. Brain.

[39] Smith SM, Jenkinson M, Woolrich MW, Beckmann CF, Behrens TE, Johansen-Berg H (2004). Advances in functional and structural MR image analysis and implementation as FSL. Neuroimage.

[40] Spencer SS (2002). Neural networks in human epilepsy: evidence of and implications for treatment. Epilepsia.

[41] Supekar K, Uddin LQ, Khouzam A, Phillips J, Gaillard WD, Kenworthy LE (2013). Brain hyperconnectivity in children with autism and its links to social deficits. Cell Rep.

[42] Tang Y, Long J, Wang W, Liao J, Xie H, Zhao G (2016). Aberrant functional brain connectome in people with antisocial personality disorder. Sci Rep.

[43] Terry JR, Benjamin O, Richardson MP (2012). Seizure generation: the role of nodes and networks. Epilepsia.

[44] Tomlinson SB, Porter BE, Marsh ED (2017). Interictal network synchrony and local heterogeneity predict epilepsy surgery outcome among pediatric patients. Epilepsia.

[45] van den Heuvel MP, Mandl RC, Stam CJ, Kahn RS, HulshoffPol HE (2010). Aberrant frontal and temporal complex network structure in schizophrenia: a graph theoretical analysis. J Neurosci.

[46] van den Heuvel MP, Sporns O (2013). Network hubs in the human brain. Trends CognSci.

[47] van den Heuvel MP, Stam CJ, Kahn RS, HulshoffPol HE (2009). Efficiency of functional brain networks and intellectual performance. J Neurosci.

[48] Vapnik V (1998). Statistical learning theory.

[49] Volz A (2008). A soviet estimate of german tank production. J Slavic Mil Stud.

[50] Wendling F, Bartolomei F, Senhadji L (2009). Spatial analysis of intracerebral electroencephalographic signals in the time and frequency domain: identification of epileptogenic networks in partial epilepsy. Philos Trans A Math Phys Eng Sci.

[51] Wilke C, Worrell G, He B (2011). Graph analysis of epileptogenic networks in human partial epilepsy. Epilepsia.

[52] Zack MM, Kobau R (2017). National and State estimates of the numbers of adults and children with active epilepsy—United States, 2015. MMWRMorb Mortal Wkly Rep.

[53] Zalesky A, Fornito A, Harding IH, Cocchi L, Yucel M, Pantelis C (2010). Whole-brain anatomical networks: does the choice of nodes matter?. Neuroimage.

[54] Zhang W, Muravina V, Azencott R, Chu ZD, Paldino MJ (2018). Mutual information better quantifies brain network architecture in children with epilepsy. Comput Math Methods Med.

[55] Zhang X, Tokoglu F, Negishi M, Arora J, Winstanley S, Spencer DD (2011). Social network theory applied to resting-state fMRI connectivity data in the identification of epilepsy networks with iterative feature selection. J Neurosci Methods.

[56] Zinkus TP (2018). Pre-surgical planning: multimodality imaging to optimize outcomes in pediatric epilepsy surgery. Mo Med.

